# Screen Printing of pH-Responsive Dye to Textile

**DOI:** 10.3390/polym14030447

**Published:** 2022-01-22

**Authors:** Marija Gorjanc, Ana Gerl, Mateja Kert

**Affiliations:** Department of Textiles, Graphic Arts and Design, Faculty of Natural Sciences and Engineering, University of Ljubljana, 1000 Ljubljana, Slovenia; marija.gorjanc@ntf.uni-lj.si (M.G.); anca.gerl@gmail.com (A.G.)

**Keywords:** pH sensitivity, flat screen printing, bromocresol green, polyamide 6, cotton

## Abstract

The development of pH-responsive textile sensors has attracted much interest in recent decades. Therefore, the aim of this study was to show that screen printing could be one of the possible techniques for development of pH-responsive textile. Several parameters that could influence the pH sensitivity and responsivity of a screen-printed textile with bromocresol green dye were studied, such as textile substrate (cotton, polyamide), printing paste composition, and type of fixation (heat and steaming). The change in mechanical and physical properties of the printed fabrics was tested according to the valid ISO, EN, or ASTM standards. The responsiveness of the printed samples to different pH values with the change in colour was evaluated spectrophotometrically. In addition, the colour fastness of the printed textiles to rubbing, washing, and light was also investigated. The results show that the textile responsiveness to pH change was successfully developed by flat screen-printing technique, which proves that the printing process could be one of the methods for the application of indicator dye to textiles. The application of the printing paste to cotton and polyamide fabrics resulted in an expected change in the mechanical and physical properties of the fabrics studied. The responsiveness of printed fabrics to the change of pH value depends on the type of fibres, the strength of dye–fibre interactions, and the wettability of the fabric with buffer solutions. The colour fastness of the printed fabrics to dry and wet rubbing is excellent. Printed polyamide fabric is more resistant to washing than printed cotton fabric. Both printed fabrics have poor colour fastness to light.

## 1. Introduction

Research on smart textiles dates to the early 1990s. Smart textiles are able to detect and respond to different stimuli from the environment, while conventional textiles offer functionality without any responsiveness to external stimuli. Due to the versatile possibilities of using smart materials, more and more research in this field is focused on the functionalization of textiles that respond to changes in the environment. Application of chromic dyes, whose response to external stimuli (UV light, temperature, pH, pressure, electric current, solvent) is governed by reversible colour change, represent an important area of passive smart textiles. In the past, colour stability was a condition, but today reversible colour change is desirable, especially for textiles that could warn people against harmful environmental conditions. Textiles that respond to different pH values by changing their colour are very useful as sensor materials. The colour signal of the textile would visually warn people about the change of the pH value in the environment [1,2,3]. In addition to the common already known electronic sensor system, textile sensors are much more suitable due to the following advantages, namely their flat shape, mechanical stability, flexibility, air permeability, washability, and reusability [4,5,6,7,8,9].

Current studies have mentioned several methods of application of pH-responsive dyes to textiles, namely conventional dyeing [5,6,10,11,12,13,14,15], spraying [16], direct incorporation of the dye during electrospinning [7,12,17,18], coating [19,20], and sol-gel technology [8,12,21], but only one study mentioned conventional printing [22]. Halochromic behaviour of pH-responsive dye is in close correlation to dye structure [10,12], presence of different substituents in dye molecule [6], fibre composition [11,12], type of dye–fibre interactions [12], fabric density [10], and application methods [12].

During conventional dyeing, leaching of the dye molecules is often noticed, therefore after-treatments of dyed fabric are recommended, which influence the halochromic behaviour of pH-indicator dye [8]. On the other hand, sol-gel technology is a more promising technology for development of pH textile sensors since no leaching is noticed [7]. However, the surrounding silica matrix affects the acid–base equilibrium of the dye due to the changing of the micro-environment [8]. Diffusion of sol into textile is in close correlation with hydrophilicity of textile. It was found out that for less hydrophilic textiles, such as polyamide, pH-responsive dye Methyl Red is present only in the outer region of the fibre, whilst in conventionally dyed fibre the dye penetrates into the core region and thus the change of colour is slower. It should be stressed that pH-responsive dyes have different halochromic behaviour in aqueous or sol solutions compared to textile or sol-gel-treated textile [8].

Incorporation of pH-indicator dyes of different concentrations into polymer PA 6.6 solution during the electrospinning process does not change electrospinning parameters and the average fibre diameter does not significantly modify [18]. Incorporation of pH-indicator dyes into polymer PA6 solution does not affect the morphology of nanofibers or the halochromic behaviour of dyes [15]. The effect of pH on fibre morphology was noticed only at pH < 1, where PA6 fibre disintegrates and release dye components into solution. At pH values from 2 to 10, only slow release of dye from PA6 in the solution was noticed, whilst the morphology of PA 6 fibre stayed intact [17]. Furthermore, the release of Bromocresol purple from PA 6.6 nanofiber is due to the lowering of the strength of dye–fibre interaction at higher pH values. The cause for this lies in the decrease of ionic interactions between anionic dye and protonated amino groups (NH_3_^+^) of PA 6.6 in the acidic medium, which became non-protonated group (NH_2_) at higher pH values [18].

In the available literature, only one study that addresses the printing of indicator dye on textiles using a conventional printing process was found [22]. The research showed that oxygen plasma treatment of PET and application of 3-propylaminotriethoxysilane (APTES) significantly increase application of two indicator dyes (Brilliant Yellow and Congo Red) using a conventional flat screen-printing technique. Printed coated fabrics showed pH responsiveness and good fastness to washing. The printing method is less time-consuming than dyeing, which according to the literature is the most common method of applying pH-responsive dyes to textiles. Moreover, water consumption for dyeing textiles accounts for 16% of the total consumption for wet processing of textiles, while water consumption for printing textiles is only 8% [23].

The purpose of the research was to study the influence of printing paste and textile substrate on the pH-responsiveness of the indicator dye bromocresol green on polyamide and cotton fabric using the flat screen-printing technique. It was assumed that the dye–fibre interaction and the hydrophilicity of the material will affect the pH response of the dye, as the colour change is strongly dependent on rapid wetting of the fabric with a buffer solution and the reaction of protonation and deprotonation of the studied dye. The mechanical and physical properties of unprinted and printed fabrics were tested as well as the colour fastness to rubbing, washing and light. The outcome of this research will be a more comprehensive knowledge of the influence of printing paste on the formation of pH-responsive textiles; thus, a less time-consuming development of textile pH-sensor can be achieved compared to conventional dyeing.

## 2. Materials and Methods

### 2.1. Fabric

In the research, chemically bleached 100% cotton fabric (CO), with a mass of 119.65 g/m^2^, density of 53 threads/cm in warp, and 30 threads/cm in weft direction, and thermofixed polyamide 6 fabric (PA6), with a mass of 75.82 g/m^2^, density 43 threads/cm in warp, and 30 threads in weft direction, were used.

### 2.2. Dye

**Figure 1:**
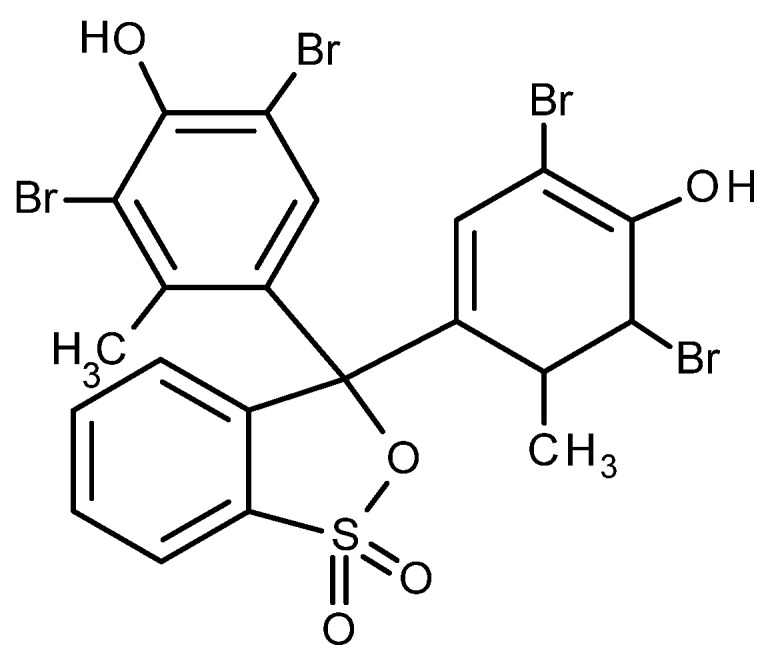
Bromocresol green.

The dye bromocresol green (BCG), a pH-sensitive triphenylmethane dye with a molecular weight of 698.01 g/mol (Honeywell Fluka, Charlotte, NC, USA), was used to print the fabrics CO and PA6. Its structural formula is shown in Figure 1. Since the dye is poorly soluble in water, it was dissolved with a small amount of ethanol before being added to the printing paste.

### 2.3. Auxiliaries

The polyacrylate thickener Clear CP, the antifoaming agent Antifoam W, the self-crosslinking binder Legante SE, the non-formaldehyde fixing agent Fixator NFO, the softner Softner A/95, and deionised water were used for the preparation of pigment printing paste. All auxiliaries are the products of Achitex Minerva, Vaiano Cremasco, Italy. Polygalactomanan ether Prisulon DCA 130 (CHT, Montlingen, Switzerland), hydrotropic agent urea (Acros Organics, Morris Plains, NJ, USA), dye-dissolving and fixing agent Glyezin A (BASF, Ludwigshafen, Germany), ammonium sulphate ((NH_4_)_2_SO_4_) (Fluka Chemie, Steinheim, Germany), and deionised water were used for the preparation of stock thickening agent for printing PA6 fabric.

### 2.4. Preparation of Printing Pastes

The compositions of the printing pastes for printing CO and PA6 samples are compiled in Table 1.

### 2.5. Printing and Aftertreatments

The cotton and polyamide fabric samples were printed with the use of electromagnetic laboratory printing machine Mini MFD R 390, (J. Zimmer, Klagenfurt am Wörthersee, Austria) using the following parameters, which differ according to the fabric composition, namely: diameter of the printing knife of 6 mm (for CO fabric) or 4 mm (for PA6 fabric); speed of the printing knife passes of 80% (for both CO and PA6 fabrics) degree of magnetic pressure of 5 (for CO fabric) or 2 (for PA6 samples); number of printing knife passes of 2 (for CO fabric) or 1 (for PA6 fabric).

The fabric samples were printed with a blank flat screen stencil, with a mesh size of 42 threads/cm (for CO fabric) or 77 threads/cm (for PA6 fabric). After printing, the samples were dried at 100 °C for 2 min. The cotton samples were then cured for 4 min at 150 °C in a laboratory dryer (W. Mathis, Oberhasli, Switzerland), while the PA6 samples were steamed normally for 20 min at 100–102 °C in a laboratory steamer DHE 20675 (Werner Mathis AG, Oberhasli, Switzerland). The wet fastness of the printed samples of PA6 was increased by cold reserving with 1.5 g/L Cibatex PA (Huntsman, Langweid am Lech, Germany) at pH 3.5–4 with 80% CH_3_COOH (Sigma Aldrich Chemie, Steinheim, Germany) for 10 min. Then the samples were rinsed with cold water, soaped with 1 g/L Tinegal W (Huntsman, Langweid am Lech, Germany) for 15 min at 50 °C and rinsed with warm and then cold water at the end.

### 2.6. Methods of Testing

#### 2.6.1. Fabric Thickness

The thickness of the unprinted and printed fabric was measured using the Metrimpex micrometre TYP 6-12-1/B (Mitutoyo Corp., Kawasaki, Japan) in accordance with the standard ISO 5084:1996 [24]. For each sample, 10 measurements of the thickness (*d*), expressed in mm, were made. The result represents an average value of 10 measurements.

#### 2.6.2. Mass per Unit Area

The mass per unit area (*W*) of unprinted and printed fabric, expressed in g/m^2^, was determined using the standard EN 12127:1997 [25]. Three measurements were taken on each sample, both unprinted and printed, and the result represents an average value.

#### 2.6.3. Stiffness

In accordance with ASTM D 1388-18 [26], the stiffness (*G*) of the tested fabrics, expressed in mg·cm, in warp (*G_w_*) and weft (*G_f_*) directions were determined according to Equation (1), as well as the total bending stiffness (*G_o_*) according to Equation (2).

(1) $$G=W{\left(\frac{O}{2}\right)}^{3}$$

(2) $$G_{o}=\sqrt{G_{w}\times G_{f}}\;$$

where *W* is the mass per surface area, expressed in g/m^2^, and *O* is the bending length, expressed in cm.

#### 2.6.4. Breaking Force, Tensile Strength and Elongation

Measurements of breaking force and elongation were made in accordance with the standard ISO 13934-1:2013 [27]. The method was modified with the measurements performed at a fasten length of 100 mm, a speed load of 50 mm/min and a specimen size of 15 cm × 2.5 cm. Seven test pieces in the warp direction and seven test pieces in the weft direction were cut from each printed sample. Each test piece was fixed in the holder of an Instron 5567 device (Instron, Buckinghamshire, UK) and then loaded. The breaking force and elongation were read at the breaking point of the test piece. The result of the measurements is the average of the measured values of breaking force (*F*) and elongation (*ε*) of the tested samples.

Tensile strength (*σ*) expressed in N/mm^2^ was calculated according to Equation (3).

(3) $$\sigma =\frac{F}{A}$$

where *F* is average breaking force of sample, expressed in N and *A* is cross-sectional surface of the sample (width x thickness), expressed in mm^2^.

#### 2.6.5. Air Permeability

The standard ISO 9237:1995 [28] was used to determine the air permeability of the fabric with an Air Tronic 3240B apparatus (Mesdan, Raffa, Italy). On unprinted and on all printed samples, 10 measurements of the air flow were made with a measuring area of 100 cm^2^ and an air pressure of 200 Pa for CO samples and 100 Pa for PA6 samples. The result of the measurements is the average value of the air flow through the sample. It should be emphasised that the printed sample was oriented with the front side facing the air opening during the measurements. From Equation (4), the air permeability (*R_p_*) of the fabric, expressed in mm/s, was calculated using an average value of the air flow $\bar{q_{v}}$ through the fabric:

(4) $$R_{p}=\frac{\bar{q_{v}}}{A}\times 167$$

where $\bar{q_{v}}$ is the air flow in l/min and *A* is the measurement area in cm^2^.

#### 2.6.6. Spectrophotometric Measurements

The colour of the printed samples was measured with a Datacolor Spectraflash 600 PLUS-CT (Lawrenceville, NJ, USA) spectrophotometer and CIELAB colour space. Measurements were made in the range 400–700 nm with a d/8° measurement geometry, under D65 illumination, 10° standard observer, with specular reflectance included and the UV component excluded (0% UV, filter FL40 on), with a 9-mm aperture. Four layers of the sample were used for the measurements. An average of 10 measurements was taken for each sample. CIELAB values were determined on unprinted and printed test pieces.

#### 2.6.7. Colour Fastness to Rubbing

Crockmeter M23888 apparatus (SDL ATLAS, Rock Hill, SC, USA) was used to test the rubbing fastness of the printed samples. The measurements were carried out in accordance with the standard ISO 105-X12:2016 [29]. The colour fastness of printed fabrics to rubbing was assessed visually using a grey scale in accordance with the standard ISO 105-A03:2019 [30].

#### 2.6.8. Colour Fastness to Washing

The standard ISO 105-C06:2010 [31] was used to determine the colour fastness of printed samples to domestic and commercial laundering. Washing was carried out in a Gyrowash machine (James Heal, Halifax, UK) using test methods A1S and A1M and ECE (European Colorfastness Establishment) detergent. The first test piece was washed with the A1S method, while the other two were washed with the A1M method. Washing with method M means that one washing cycle is equivalent to five domestic washing cycles. The third test piece was washed twice with the A1M method, which corresponds to 10 domestic washing cycles. Washing was done at 40 °C and lasted 30 min for the A1S method and 45 min for the A1M method. After rinsing and drying, colour fastness to washing was visually assessed using a grey scale according to the standard EN 20105-A02:1994 [32] for colour change and the standard ISO 105-A03:2019 [29] for staining of the adjacent fabric.

#### 2.6.9. Colour Fastness to Light

The printed samples were subjected to the standard ISO 105-B02:2014 [33] in a Xenotest Alpha device (Atlas, Rancho Cucamonga, CA, USA). A test piece of each printed sample was illuminated for only 4 h under the following conditions: 70% relative humidity in the test chamber, 42 W/m^2^ power of irradiance from a xenon lamp, a temperature of 35 °C in the chamber, and a temperature of 50 °C for the black standard. The colour difference (∆E*_ab_), calculated between samples before and after illumination, was used to evaluate light fastness. A higher difference means a lower light fastness of the printed fabric.

#### 2.6.10. Determination of pH Response of Printed Samples to Colour Change

Colour response of printed fabrics to pH was determined after immersing the test pieces in buffer solutions with different pH values, namely 3, 4, 5, 6, 7, 9 and 11. After 3 min, all test pieces were removed from the buffers, dried in air, and evaluated spectrophotometrically.

#### 2.6.11. Determination of Response Time

After the test piece of printed fabric had been dipped into the buffer solution, the time (t) at which the tested fabric showed a change in colour was determined.

## 3. Results and Discussion

### 3.1. Results of the Mechanical and Physical Properties of the Fabric

Table 2 summarises the results of the mechanical and physical properties of unprinted and printed cotton (CO) and polyamide 6 (PA6) fabrics. The results show that the application of printing paste leads to an increase in fabric thickness of 10.5% for CO and 0.53% for PA6. The mass per surface area (W) of CO fabric increases by 18.6%, but only by 3.3% for PA6 fabric. The higher increase in W for CO fabric was attributed to the ingredients of the printing paste, all of which remain on the cotton fabric, as the printed fabric was not post-treated after curing, which could remove the ingredients from the fabric. A much lower increase in W was observed for printed PA6 fabric. The latter was attributed to both dye fixation and an increase in fabric thickness due to 20 min of normal steaming of the printed fabric, as well as the removal of the unfixed dye and all other ingredients of the printing paste from the PA6 fabric during the after-treatments. In addition, the stiffness of both fabrics changed drastically after printing, both in the warp and weft directions. It should be emphasised that both fabrics were printed over the entire surface, which is why the stiffness of the fabric increased by 71% in the warp and 90% in the weft direction for CO and by 45% in the warp and 35% in the weft direction for PA6. The reason for the lower increase in fabric stiffness of PA6 fabric compared to CO fabric is again due to the presence of all ingredients in the printing paste, which remain on CO fabric after printing, compared to PA6 fabric, where all chemicals (urea, Glyezin A, ammonium sulphate and thickener) were removed during the after-treatment processes.

Breaking force as well as tensile strength of the samples examined is higher in the warp direction than in the weft direction, regardless of the fabric composition. The reason for this is the higher density of the warp threads in both CO and PA6 fabrics. The tensile strength is higher for PA6 fabric compared to CO fabric, both in the warp and in the weft directions. The latter is to be expected as the strength of a fabric depends on the yarn structure, strength of the yarn in the warp and weft direction, the bending behaviour of the yarn, the weave, and the fabric geometry. After applying the printing paste, the breaking force of CO fabric increases in the warp direction, while it remains practically unchanged in the weft direction. In contrast to the breaking force, the tensile strength of the fabric CO decreases in both directions after printing, which means that the strength of the fabric CO decreases after printing. The breaking force as well as tensile strength of printed PA6 fabric decreases in the warp direction and increases in the weft direction compared to unprinted PA6 fabric. PA6 fabric was found to shrink during steaming, which was confirmed by determining the percentage shrinkage of PA6 fabric during fixation, which was 1.87% in the warp direction and 1.06% in the weft direction. The values of elongation at break are slightly higher in the weft direction than in the warp direction, regardless of the fabric composition. The latter is a consequence of the lower tension of the weft threads and the higher tension of the warp threads during the weaving process. The elongation at break is almost not changed after printing, except for PA6 fabric in the warp direction, where an increase in elongation is observed. The elongation at break of the fabric depends on the fibre composition, the yarn structure in both directions, the weave and the yarn bending. To fully understand the tensile behaviour of the tested fabrics after printing, further tests should be carried out, but this is beyond the scope of the present study.

The air permeability of the fabrics decreased after printing, but to a greater extent for CO than for PA6 fabrics. The reason for this is the formation of a bonding layer on the CO fabric during curing, which causes fibre bonding and the closure of the free spaces between warp and weft threads. PA6 fabric was found to have a smaller increase in air permeability, which was attributed to the shrinkage of the fabric after the printed fabric was fixed by normal steaming.

### 3.2. Colour and Colour Fastness

**Figure 2:**
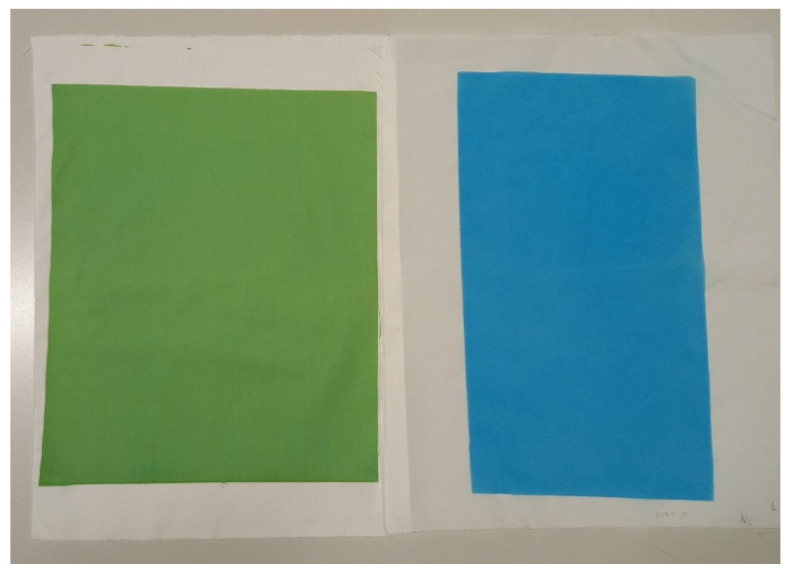
Printed CO fabric (**left**) and PA6 fabric (**right**).

Table 3 shows that after printing, the fabric CO became darker (the value of CIE L* decreases), greener (the value of CIE a* decreases and is negative), and yellower (the value of CIE b* increases and is positive). Compared to the cotton fabric, the polyamide fabric became darker after printing (the value of CIE L* decreases) and bluer (the value of CIE b* is lower and negative), while the value of CIE a* is the same as the value of CIE a* of the printed CO fabric. The colour of the printed PA6 fabric is more saturated (higher value of C*_ab_) in contrast to CO. The colours of the printed samples are shown in Figure 2.

Printed CO fabric is green–yellow, while PA6 fabric is blue. Several factors can influence the colour of the fabric. The pH of the pigment printing paste is 8, but the pH of the printing paste for printing PA6 is 5.5. At a pH above 5, the BCG dye is in dianionic form and the colour of the dye is blue [6,34]. Visually, both prepared printing pastes were blue in colour. During curing, the pH of the pigment printing paste applied to CO changes and becomes more acidic, which is due to the formation of a binder layer where the thickener acts as a catalyst and accelerates the cross-linking of the binder. In addition, the BCG dye is protonated and changes colour from blue to green–yellow. Unlike CO, the PA6 fabric remains blue when steamed. It is assumed that the BCG dye in dianionic form has no affinity to the negatively charged cellulose fibres where only electrostatic repulsive forces are present, which means that there are no dye–fibre interactions. In contrast, the BCG dye in dianionic form forms electrostatic attractive forces with positively charged NH_3_^+^ groups of the PA6 fibres, which is due to the protonation of the amino groups (−NH_2_) of PA 6 during steaming. The protonation occurs in an acidic medium due to the presence of tartaric acid in the printing paste.

**Figure 3:**
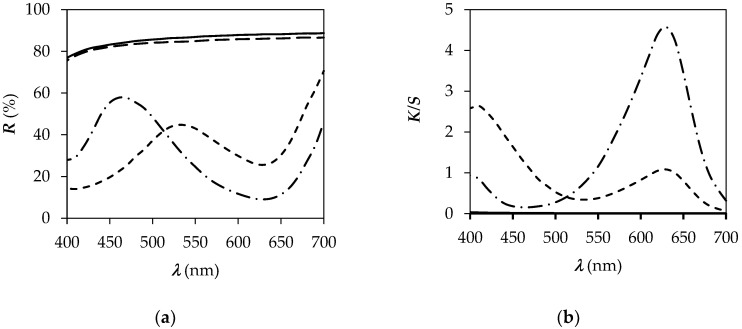
Values of *R* (**a**) and *K/S* (**b**) vs. wavelength (*λ*) for unprinted (CO and PA) and printed (CO_P and PA6_P) samples. — CO. - - - CO_P. – – PA6. –·– PA6_P.

The reflectance curves of unprinted and printed CO and PA6 fabrics are shown in Figure 3. The reflectance curves of the fabrics changed significantly after printing, which is due to the presence of dye on the fabric. The *K/S* values versus wavelength shown in Figure 3 clearly indicate that the colour strength of the printed CO fabric at a wavelength of 630 nm is much lower than that of the PA6 fabric and at 410 nm is higher for CO fabric than PA6 fabric. The pH of the printing paste is also believed to affect the colour achieved on the fabric and the way the dye binds to the fibres. From the CIELAB values in Table 3, it is clear that the printed CO fabric is lighter and yellow green (the value of CIE a* is negative and the value of CIE b* is positive), while the printed PA6 fabric is darker and blue–green (both values of CIE a* and CIE b* are negative).

**Figure 4:**
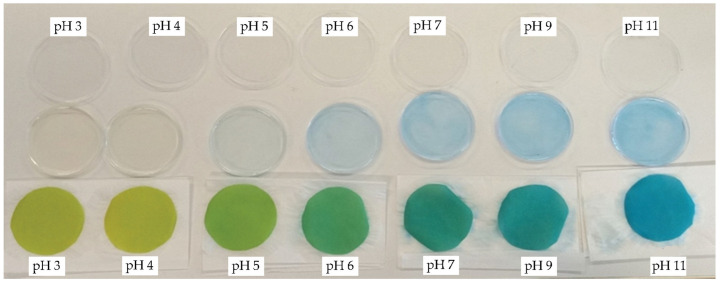
CO_P samples after immersion into buffer solutions of pH 3, 4, 5, 6, 7, 9 and 11 (from **left** to **right**).

After immersing the printed samples in buffer solutions with different pH values, the pH sensitivity of the sample was observed. It should be emphasised that the pH sensitivity of the indicator dye is influenced by several factors, namely the wetting of the fabric with the buffer solution, the position of the dye in the fibre, the strength of the interactions between the dye and the fibre and the post-treatment procedure. It is therefore difficult to say that one factor outweighs the other, but the pH sensitivity of the indicator dye results from the sum of the individual factors. Visually, it was found that the fabrics from CO reacted more quickly to the change in pH than the PA6 fabric. The higher hydrophilicity of the CO fabric and thus a stronger wetting of the fabric with the buffer solution in contrast to the PA6 fabric is reflected in its faster response. The same results were found by van den Shueren and De Clerck [10]. It was also hypothesized that in the case of pigment printing paste, the BCG dye would remain on the fabric surface, entrapped in the binder layer and therefore could respond more quickly to the change in pH, compared to the BCG dye in PA 6 fabric, which penetrates deeper into the fibres during steaming and forms dye–fibre interactions; therefore, its ability to respond to the change in pH would be less pronounced or even compromised. The indicator dye located in the outer region of the fibre (i.e., in the sol-gel-treated PA6 fabric) was found to respond more rapidly to the change in pH than the dye that penetrates into the core region of the PA 6 fabric during dyeing [8]. Usually, after applying pigment-printing paste, no post-treatment of the fabric is required, whereas when printing polyamide, rinsing to remove thickener, unfixed dye, and the remaining components of the printing paste is strongly recommended. Typically, post-treatment of polyamide ends with treatments to improve the wash fastness properties of the printed fabric. Published results show [10,34] that the cationic after-treatment of cotton fabric and the oil and water repellent finish of polyamide 6 fabric dyed with indicator dye affect the pH sensitivity of the fabric. Thus, it could be assumed that the post-treatment of printed PA6 in our study could influence the pH sensitivity of the fabric. Further research should be conducted to confirm or reject this assumption, which is not within the scope of the current research. The response time was less than 30 s for CO fabric and more than 2 min for PA6 fabric. As the BCG dye remains poorly soluble in the pigment printing paste and therefore small spots of undissolved dye could be seen on the printed fabric, the dye was dissolved in ethanol before being added to the printing paste. After immersing the CO_P samples in buffer solutions, bleeding of the BCG dye into the buffer solutions was observed (Figure 4).

The latter was attributed to the fact that the BCG dye does not make any attractive interactions with the cotton fabric when the dye is in soluble form. Since the dye is negatively charged due to the presence of sulphonic group, it could not bind to negatively charged cotton fibres. It was also assumed that the binder is not able to bind the BCG dye to the textile fibres, as is the case with pigments, which are insoluble colour particles that are larger than the dye and have no affinity to textile fibres. It can be seen from Table 4 that the lightness of the CO_P samples decreases after immersing the samples in buffer solutions with a pH of 5–11, which means that the samples become darker. The values of CIE a* were negative and decreased, while the values of CIE b* decreased and changed from a negative to a positive value. This means that the CO samples became greener, less yellow and bluer as the pH value increased from 5 to 11.

**Figure 5:**
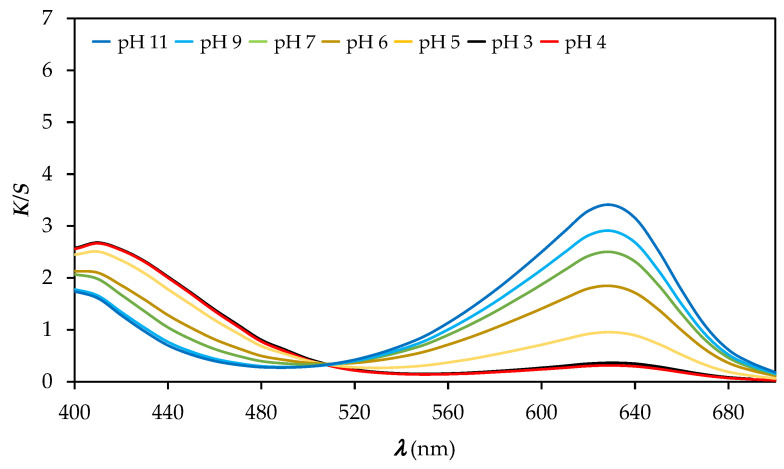
Values of *K/S* vs. wavelength (*λ*) of printed CO_P samples immersed into buffer solution of different pH values.

Figure 5 shows the values of *K/S* as a function of the wavelength of the CO_P samples after immersion in buffer solutions with different pH values. It can be seen from Figure 5 that when the pH is decreased from pH 11 to pH 3, the *K/S* values at 630 nm decrease, but when the pH is increased from pH 3 to pH 11, the *K/S* values at 410 nm decrease. The printed cotton samples turned yellow at pH 3 and 4 and gradually changed to a blue–green hue at higher pH values. The latter indicates that the printed fabric reacts with the change of colour due to change of pH values, as shown in Figure 5.

**Figure 6:**
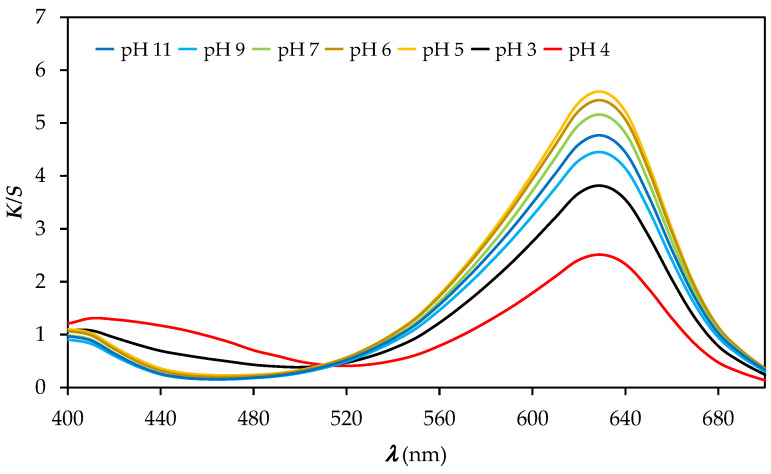
Values of K/S vs. wavelength (λ) of printed PA6_P samples immersed into buffer solution of different pH values.

**Figure 7:**
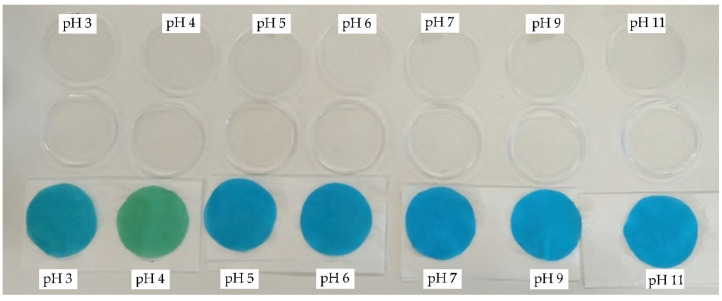
PA6_P samples after immersion into buffer solutions of pH 3, 4, 5, 6, 7, 9 and 11 (from **left** to **right**).

The *K/S* values of the PA_P samples are lowest at 630 nm and highest at 410 nm in buffer solutions with pH 3 and 4, as shown in Figure 6. The printed PA6 fabric responds to pH and the colour change can be visually detected, but the colour change is less obvious (Figure 7) than for the printed cotton fabric (Figure 4). The printed PA6 samples did not bleed after immersion in buffer solutions of different pH values, indicating the binding of the BCG dye to the PA6 fibres through ionic interactions formed between the anionic sulfonic group of the BCG dye and the protonated amino groups of the PA6 fibres. The CIELAB values for the PA6_P samples compiled in Table 4 show that the sample at pH 4 is yellow–green and brighter than the samples of PA6_P at pH values of 3, 5, 6, 7, 9 and 11. After immersing the samples in buffer solutions with pH values ranging from pH 6 to pH 11, the samples were found to be darker, less green, and bluer than the samples immersed in buffer solution with pH 4. Moreover, no obvious changes in *K/S* values at 410 nm are seen at pH values from pH 5 to pH 11, while the decrease in *K/S* values is observed with the increase in pH value from pH 5 to pH 11. It was concluded that both the nature of the dye–fibre interactions and the strength of the dye–fibre interactions affect the pH response of the printed PA6 fabric to the change of colour. Similar results were noticed by Giachet et al. and De Clerck et al. [5,12].

Assessments of colour fastness properties of studied samples to washing, rubbing and light, are gathered in Table 5, Table 6 and Table 7. They showed that both printed fabrics had excellent colour fastness to dry and wet rubbing.

The staining of adjacent fabrics in colour fastness to washing was not found, but a change in colour of the cotton samples was noticed after 1, 5 and 10 washing cycles, implying that the cotton samples did not have good resistance to washing. The latter was to be expected as the dye is not bound to the fibre by a bonding layer as is the case with pigments. Both the cotton and polyamide 6 printed samples have very poor light fastness which was expected, since BCG dye belongs to triphenylmethane dyes for which the poor lightfastness properties is significant [35]. After exposing the samples to the Xenotest device for only 4 h, the colour difference between unexposed and exposed samples was 17.36 for cotton and 31.08 for polyamide 6, which means that the fading of the colour could be perceived by the naked eye.

## 4. Conclusions

The research results show that the pH-sensitive textile could be successfully produced by applying BCG dye to CO and PA6 fabric using the flat screen-printing technique. Printing can be one of the techniques used for the application of BCG dye to the textile substrate.

Changes in mechanical and physical properties due to the application of the printing paste were observed for both printed fabrics. The increase in mass per surface area, thickness and stiffness was observed in both printed fabrics, but the increase was higher for cotton than for polyamide 6 fabric, which is due to the presence of all the ingredients of the printing paste on the cotton fabric and their removal from the polyamide fabric during after-treatment processes. The breaking strength of the tested samples is higher in the longitudinal direction than in the transverse direction. The tensile elongation is minimally higher in the direction of the weft threads compared to the warp threads. The formation of a bonding layer on the cotton fabric, which causes the fibres to stick together and close the empty spaces between the warp and weft threads, significantly reduces the air permeability. A much smaller reduction in the air permeability of polyamide fabric is due to the shrinkage of the fabric during normal steaming.

The colour response of the printed fabric to the change in pH depends on the fabric composition and the corresponding ability of the fabric to wet after immersion into buffer solutions. The colour depth was higher for PA6 fabric than for CO fabric. For printed CO fabric, the colour change due to the change in pH of the buffer solutions was visually noticeable, whereas for PA6 fabric, the colour change was present, but less noticeable.

The evaluation of colour fastness shows that both fabrics tested have excellent resistance to dry and wet rubbing, while the wash resistance of the CO fabric was poor, but the staining of the adjacent fabrics was not detected, due to repulsive forces between BCG dye and CO fabric.

Further research should be focused to application of BCG dye to cotton fabric using more suitable printing paste which will assure excellent adhesion of BCG dye to cotton fabric without deterioration of fabric wetting and with improved washing and light fastness.

## Figures and Tables

**Table 1:** Composition of printing pastes.

Printing Paste for Printing of CO Fabric		Printing Paste for Printing of PA6 Fabric	
Concentration (g/kg)	Component	Concentration (g/kg)	Component
1	dye BCG	1	dye BCG
3	ethanol	3	ethanol
729	H_2_O	100	urea
3	Antifoam W	50	H_2_O, cold
34	Clear CP	236	H_2_O, boiling
200	Legante SE	50	Glyezin A
10	Softner A/95	500	Prisulon DCA 130 13%
20	Fixator NFO	60	(NH_4_)_2_SO_4_ (1:2)

**Table 2:** Thickness (*d*), mass per surface area (*W*), stiffness (*G*), breaking force (*F*), tensile strength (*σ*), elongation at break (*ε*), and air permeability (*R*_p_) of unprinted (CO and PA6) and printed (CO_P and PA6_P) samples.

Sample	*d* (mm)	*W* (g/m^2^)	*G* (mg × cm)			*F* (N)		*σ* (N/mm^2^)		*ɛ* (%)		*R_p_* (mm/s)
			*G_w_*	*G_f_*	*G_o_*	Warp	Weft	Warp	Weft	Warp	Weft	
CO	0.2321	119.65	271.55	114.81	176.57	376.96	211.57	64.97	36.46	12.13	13.24	442.67
CO_P	0.2564	141.86	463.49	217.53	88.28	384.33	210.27	59.96	32.80	12.04	13.92	37.32
PA6	0.1318	75.82	94.91	82.12	317.52	366.38	296.14	111.19	89.88	38.21	46.06	344.19
PA6_P	0.1325	78.30	137.62	110.80	123.48	342.81	320.82	103.49	96.85	42.62	47.80	210.12

**Table 3:** CIELAB colour values, chroma (C*_ab_) and hue (*h*) of unprinted (CO and PA6) and printed (CO_P and PA6_P) samples.

Sample	L*	a*	b*	C*_ab_	*h_ab_* (°)
CO	94.63	−0.14	2.70	2.70	92.95
CO_P	67.19	−19.74	23.80	30.91	129.67
PA6	93.84	−0.09	2.20	2.20	92.43
PA6_P	59.23	−20.93	−29.93	36.52	235.03

L* lightness, a* red–green coordinate in CIELAB colour space, b* yellow–blue coordinate in CIELAB colour space.

**Table 4:** CIELAB colour values. chroma (C*_ab_) and hue (*h*) of printed (CO_P and PA6_P) samples after immersion into buffer solution of different pH values.

Sample	pH	L*	a*	b*	C*_ab_	*h_ab_* (°)
CO_P	3	75.83	−12.30	38.80	40.70	107.60
	4	76.67	−11.61	39.72	41.38	106.30
	5	69.52	−21.55	24.67	32.76	131.14
	6	64.13	−26.11	7.77	27.24	163.43
	7	62.32	−28.18	−0.21	28.18	180.43
	9	61.89	−28.06	−7.64	29.08	195.23
	11	60.85	−28.48	−11.18	30.60	201.43
PA6_P	3	59.14	−26.73	−11.16	28.97	202.65
	4	62.08	−26.33	5.43	26.93	168.34
	5	57.02	−23.32	−27.50	36.07	229.69
	6	57.42	−22.35	−29.05	36.65	232.43
	7	58.47	−21.47	−30.87	37.60	235.18
	9	59.54	−20.87	−29.60	36.22	234.82
	11	58.75	−20.87	−30.17	36.69	235.32

**Table 5:** Colour fastness to washing.

Sample	Number of Washing Cycles	Visual Assessment Using Grey Scale		
		Staining of 1st Adjacent Fabric	Staining of 2nd Adjacent Fabric	Colour Change
CO_P	1	5	5	3
	5	5	5	2/3
	10	5	5	2
PA6_P	1	5	5	5
	5	5	5	5
	10	5	5	5

**Table 6:** Colour fastness to rubbing.

Sample	Dry Rubbing		Wet Rubbing	
	Warp	Weft	Warp	Weft
CO_P	5	5	5	2/3
PA6_P	5	5	5	2/3

**Table 7:** CIELAB colour values, chroma (C*_ab_), hue (*h_ab_*) of printed (CO_P and PA6_P) samples after illumination and colour difference (∆E*_ab_).

Sample	L*	a*	b*	C*_ab_	*h_ab_* (°)	∆E*_ab_
CO_P	78.69	−6.73	23.34	24.30	106.10	17.36
PA6_P	78.99	−16.21	−6.41	17.44	201.49	31.08

## Data Availability

Not applicable.
